# Supplementary figures and images for: The oxidative stress response of pathogenic Leptospira is controlled by two peroxide stress regulators which putatively cooperate in controlling virulence
Source: PLoS Pathog. 2021 Dec 2;17(12):e1009087. doi: 10.1371/journal.ppat.1009087 (PMC8638851; doi:10.1371/journal.ppat.1009087)

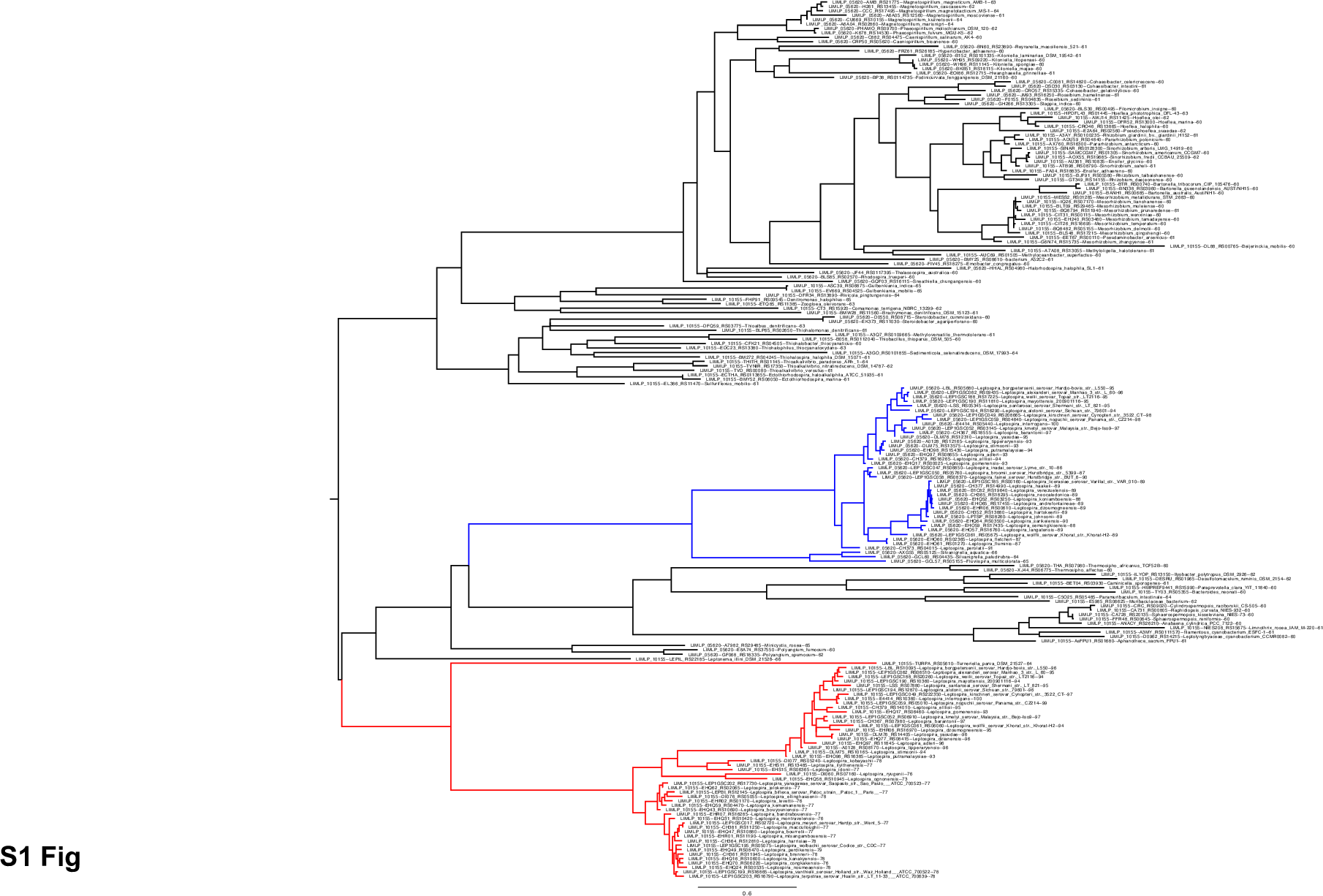

Supplement: S1 Fig — Extended phylogenetic tree showing the separation between PerRA (LIMLP_10155 in red) and PerRB (LIMLP_05620 in blue). (TIF) [file ppat.1009087.s001.tif]

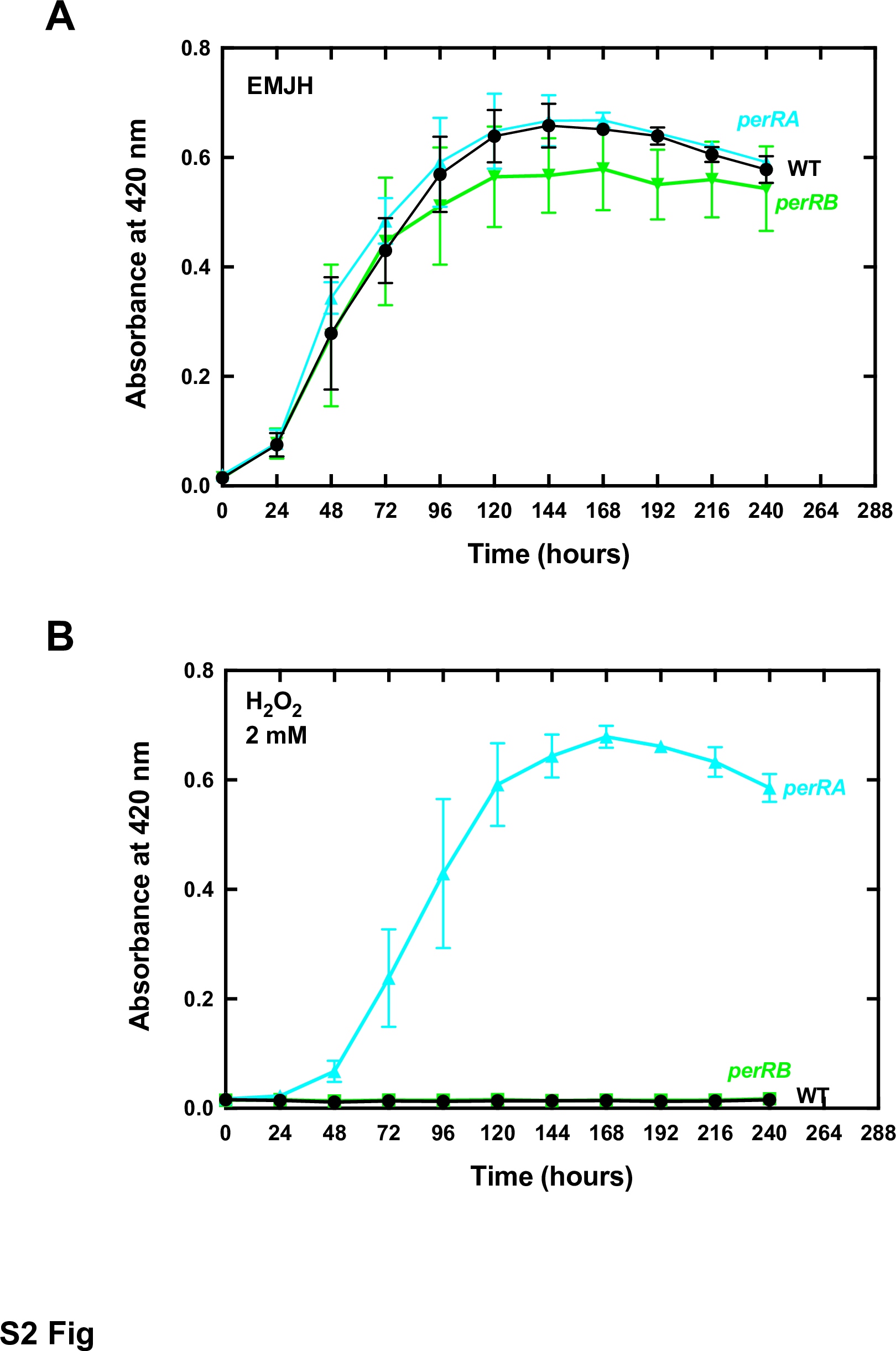

Supplement: S2 Fig — L. interrogans WT (black circles), perRA (cyan triangles) and perRB (green inverted triangles) mutant strains were cultivated in EMJH medium at 30°C in the absence (A) or presence of 2 mM H2O2 (B). Leptospira growth was assessed by absorbance at 420 nm. Data are means and standard errors of three independent biological experiments. (TIF) [file ppat.1009087.s002.tif]

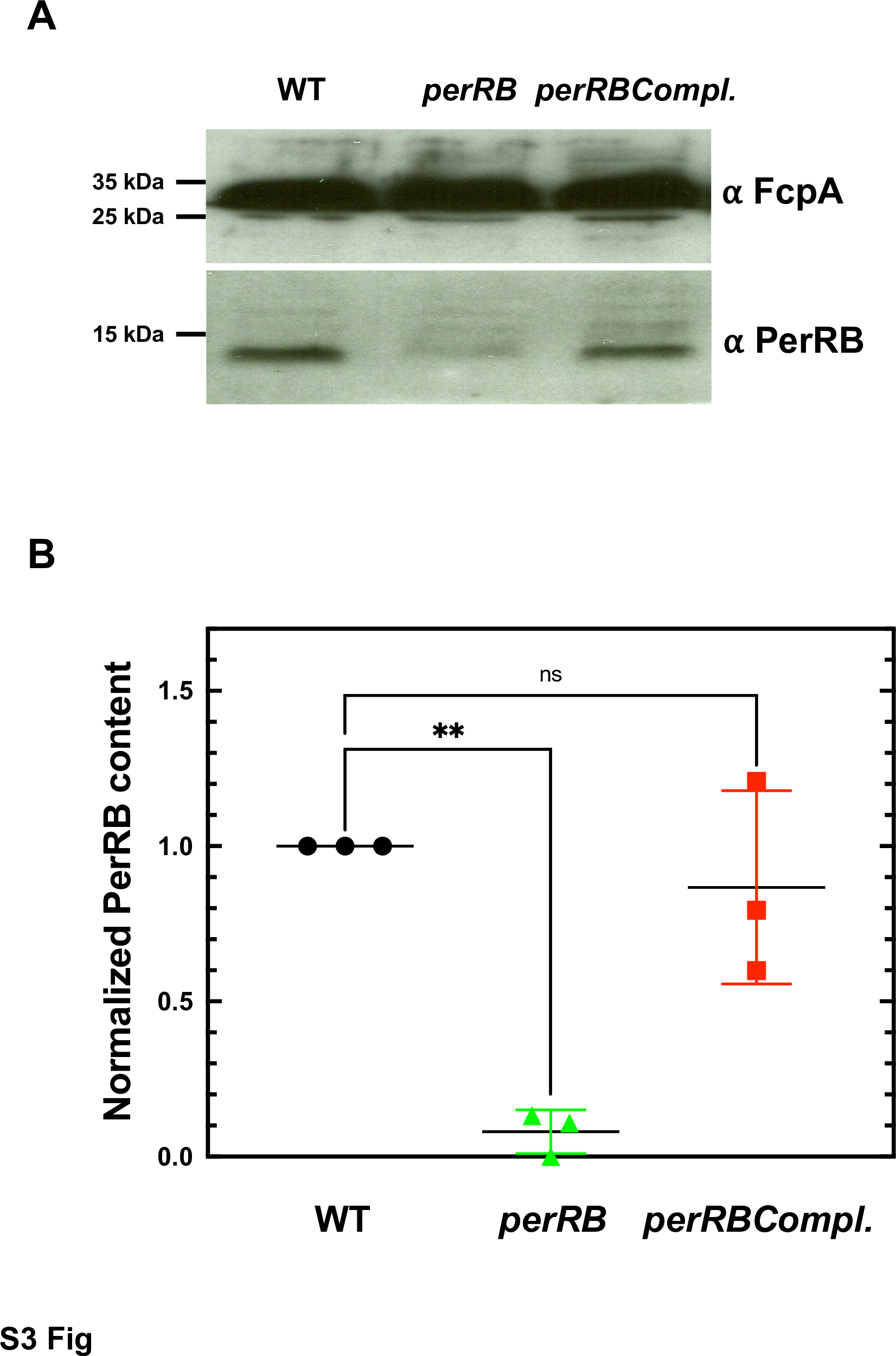

Supplement: S3 Fig — L. interrogans WT, perRB mutant and perRB mutant complemented strains were cultivated in EMJH medium at 30°C until the logarithmic phase and lyzed by sonication in 25 mM Tris pH 7.5, 100 mM KCl, 2 mM EDTA, 5 mM DTT, with a protease inhibitors cocktail (cOmplete Mini EDTA-free, Roche). 10 μg of total lyzates were resolved on a 15% SDS-PAGE and transferred on nitrocellulose membrane. PerRB was detected by immunoblot (A) using a rabbit polyclonal antibody at 1/500 dilution. FcpA production was assessed as a control of equal loading using a rabbit polyclonal antibody at 1/1000 dilution. A goat anti-rabbit IgG secondary antibody coupled to the HRP peroxidase (Sigma) was used at a 1/150000 dilution. Detection was performed by chemiluminescence with the Supersignal West Pico PLUS reagent (ThermoScientific). PerRB content was quantified using ImageJ (Schneider et al., Nat Methods 9, 671–675 (2012) https://doi.org/10.1038/nmeth.2089) and normalized by the quantity in the WT strain (B). Data are means and standard errors of three independent biological experiments. Statistical significance was determined by a One-way Anova test in comparison with WT samples (**, p-value = 0.0018). (TIF) [file ppat.1009087.s003.tif]

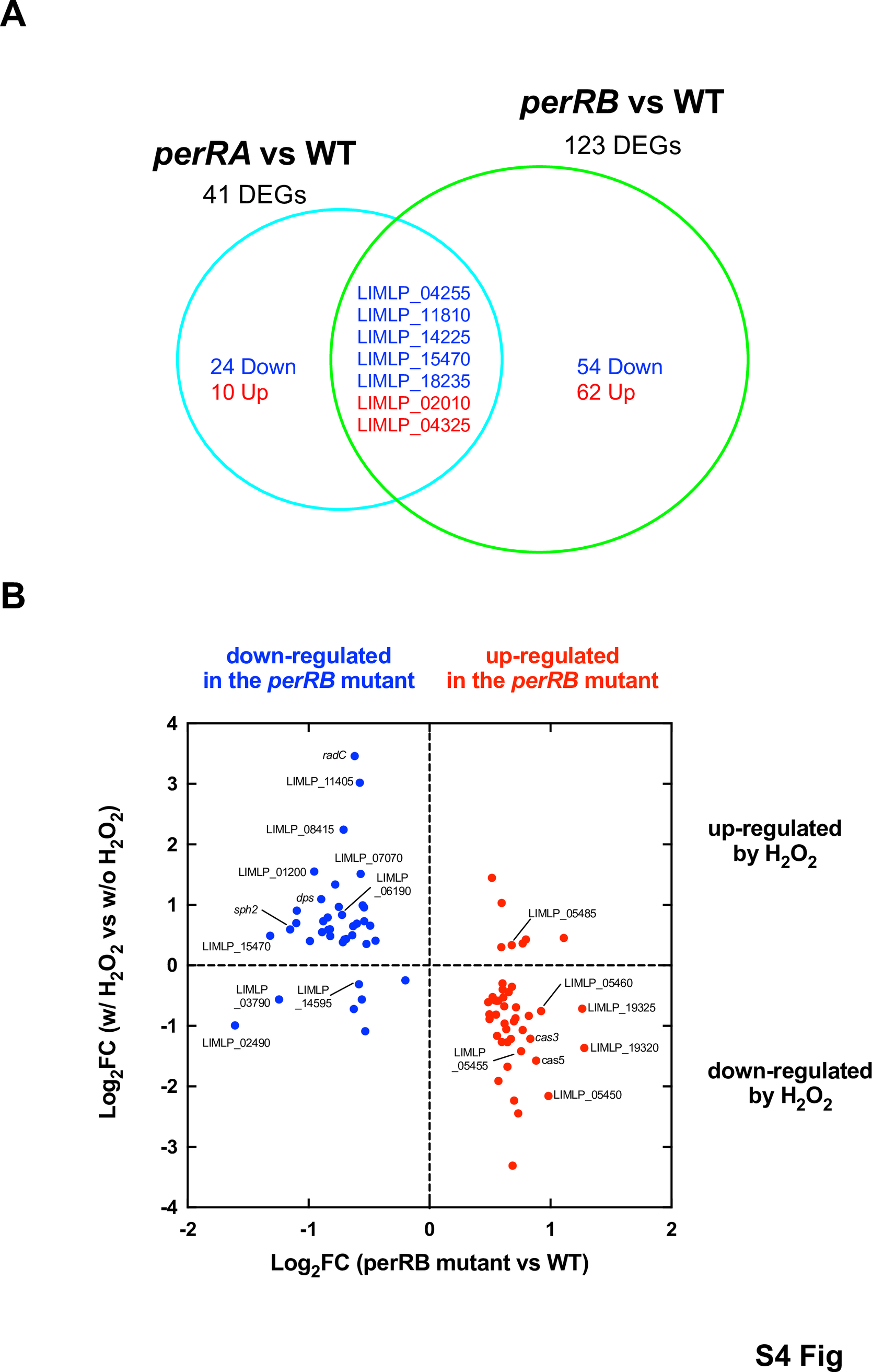

Supplement: S4 Fig — (A) Venn diagram showing the overlap of differentially-expressed ORFs (with an adjusted p-value < 0.05) in the perRA and perRB mutants. Differentially-expressed genes in the perRB mutant (as determined in this study) (in green) were compared with those in the perRA mutant as determined previously (Zavala-Alvarado et al., PLOS Pathogens. 2020 Oct 6;16(10):e1008904) (in cyan). The down- and up-regulated ORFs in both mutants were indicated in blue and red, respectively. (B) Comparison of differentially-expressed ORFs (with an adjusted p-value < 0.05) in the perRB mutant and upon L. interrogans exposure to H2O2. Log2FC of differentially-expressed ORFs upon L. interrogans exposure to 1 mM H2O2 (as determined previously (Zavala-Alvarado et al., PLOS Pathogens. 2020 Oct 6;16(10):e1008904) was plotted against the Log2FC of differentially-expressed ORFs upon perRB inactivation. Down- and up-regulated ORFs in the perRB mutant were represented by blue and red symbols, respectively, and the name of selected ORFs was indicated. The dashed lines indicate a Log2FC value of zero. Please note that only differentially-expressed ORFs in both conditions were considered. (TIF) [file ppat.1009087.s004.tif]

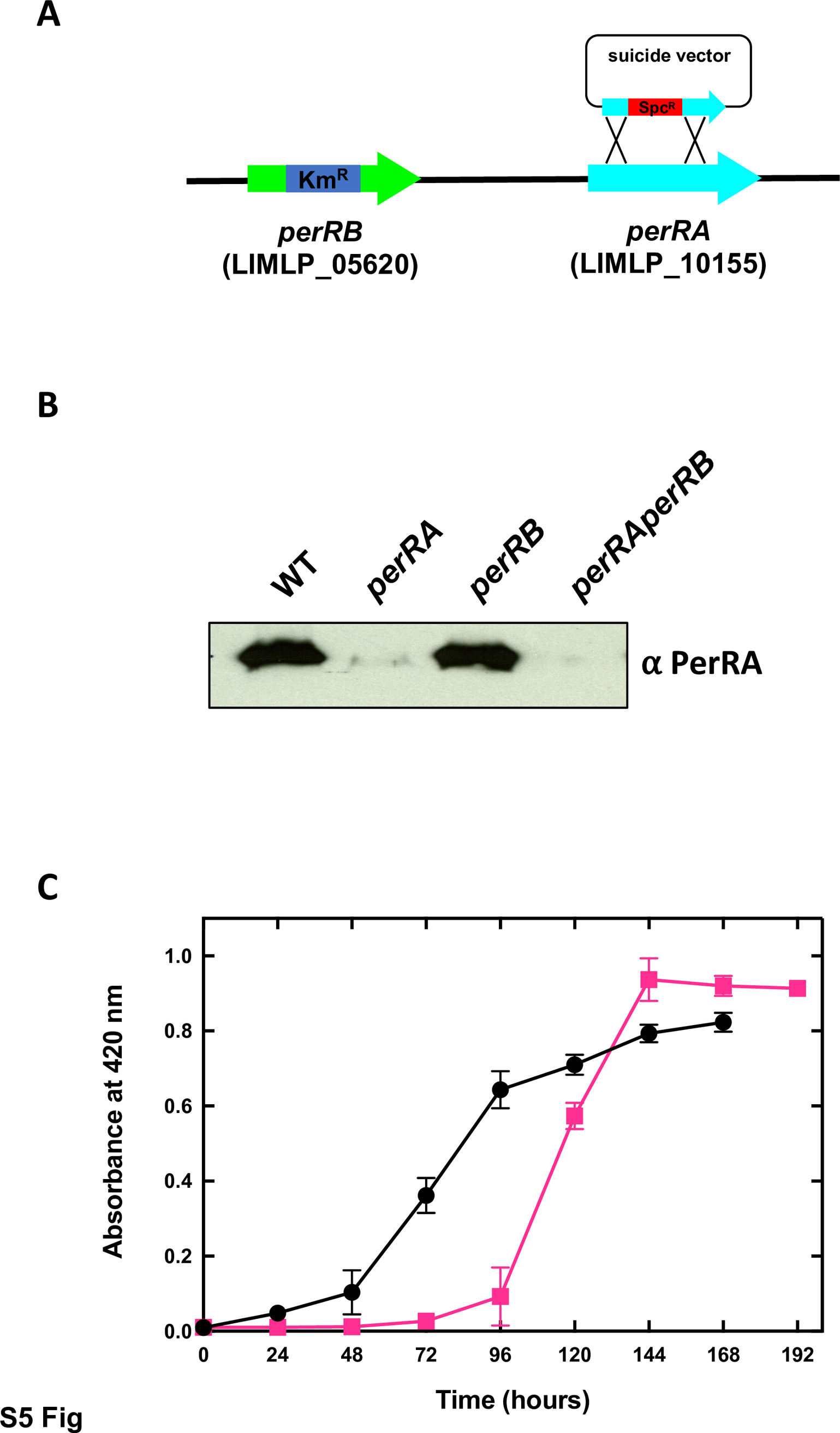

Supplement: S5 Fig — (A) Schematic representation of the double perRAperRB mutant construction. PerRA (LIMLP_10155) was inactivated by allelic exchange in the transposon perRB mutant. The kanamycin (Km) and spectinomycin (Spc) resistance cassettes inactivating perRB and perRA, respectively, are indicated. (B) Production of PerRA in the WT, in the single perRA and perRB mutants and in the double perRAperRB mutant strains. L. interrogans strains were cultivated in EMJH medium at 30°C until the logarithmic phase and lyzed by sonication in 25 mM Tris pH 7.5, 100 mM KCl, 2 mM EDTA, 5 mM DTT, with a protease inhibitors cocktail (cOmplete Mini EDTA-free, Roche). 10 μg of total lyzates were resolved on a 15% SDS-PAGE and transferred on nitrocellulose membrane. PerRA was detected by immunoblot using a 1/2000 antibody dilution as described previously (Kebouchi et al., J Biol Chem. 2018;293(2):497–509. doi:10.1074/jbc.M117.804443). (C) Growth of stationary phase-adapted WT and perRAperRB mutant strains. L. interrogans WT (black circles) and perRAperRB mutant (pink squares) were cultivated in EMJH medium at 30°C until late stationary phase (7 days after the entry in the stationary phase) and used to inoculate EMJH medium. Bacteria were then cultivated at 30°C and growth was assessed by absorbance at 420 nm. Data are means and standard errors of three independent biological experiments. (TIF) [file ppat.1009087.s005.tif]

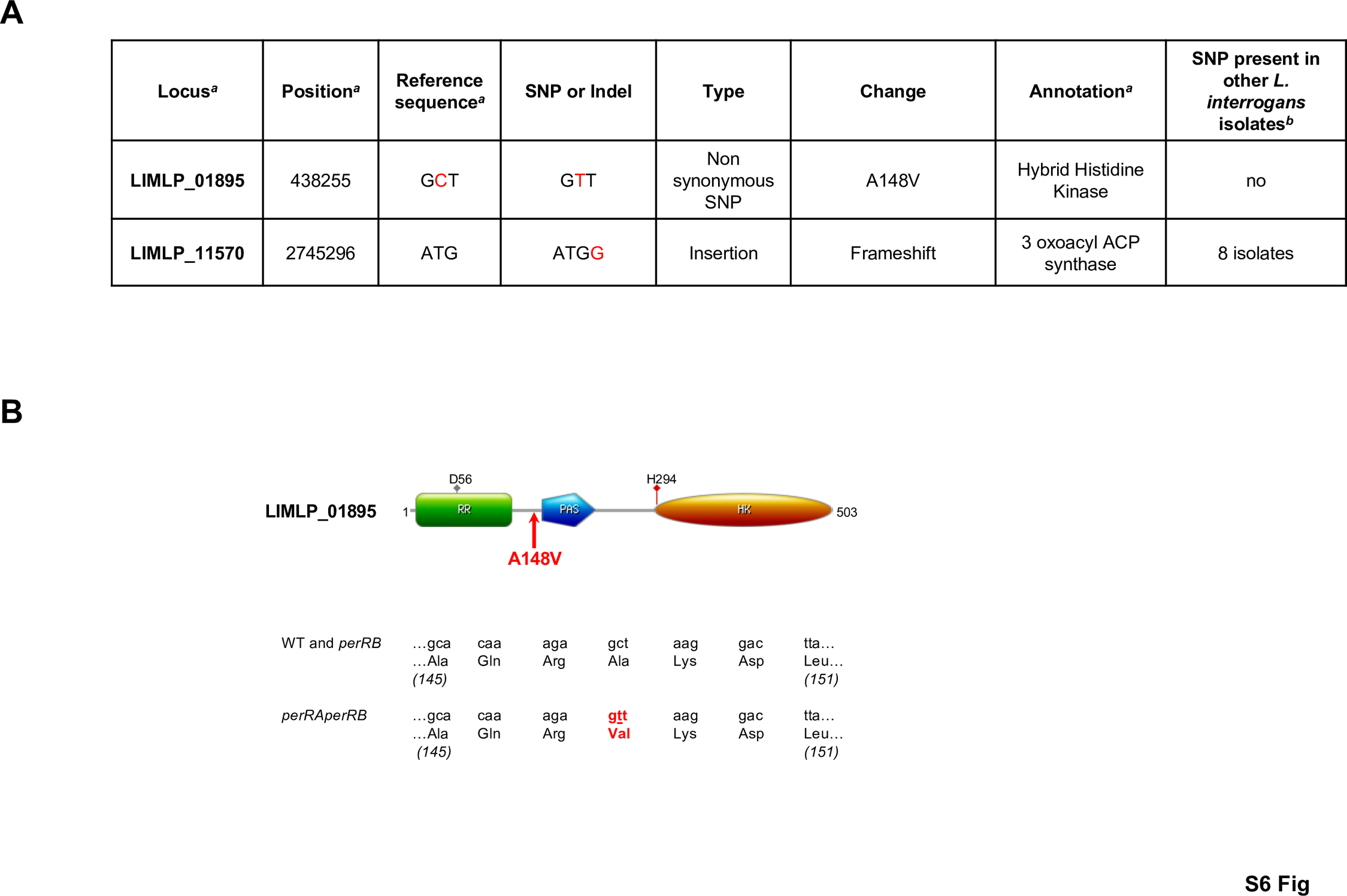

Supplement: S6 Fig — (A) Genomic DNA of perRB mutant and perRAperRB mutant strains was extracted with the Maxwell 16 cell DNA purification kit (Promega) and sequenced by Next-generation sequencing. Sequence Reads were processed by fqCleaner and aligned with the reference sequenced genome of Leptospira interrogans serovar Manilae strain UP-MMC-NIID LP (accession numbers CP011931, CP011932, CP011933; Satou et al., Genome Announc. 2015 Aug 13;3(4):e00882-15. doi: 10.1128/genomeA.00882-15. PMID: 26272567; PMCID: PMC4536678.) using Burrows-Wheeler Alignment tool (BWA mem 0.7.5a) (Li & Durbin, Bioinformatics. 2009;25(14):1754–1760. doi:10.1093/bioinformatics/btp324). SNP and Indel calling was done with the Genome Analysis Tool Kit GATK2 following the Broad Institute best practices (McKenna et al., Genome Res. 2010;20(9):1297–1303. doi:10.1101/gr.107524.110). The mutations identified (indicated in red) were further confirmed by sequencing PCR-amplified DNA fragments. 358 L. interrogans genomes of a cgMLST Leptospira isolates database (https://bigsdb.pasteur.fr/leptospira/) (Guglielmini et al., PLoS neglected tropical diseases, (2019) 13(4), e0007374. https://doi.org/10.1371/journal.pntd.0007374) were screened for homolog of the affected ORFs using BLASTN 2.9.0+ (Altschul et al., Nucleic Acids Res. 1997;25(17):3389–3402. doi:10.1093/nar/25.17.3389). Identified mutations were searched by alignment using MAFFT version 7.453 (Katoh & Standley, Molecular Biology and Evolution, Volume 30, Issue 4, April 2013, Pages 772–780, https://doi.org/10.1093/molbev/mst010). a Positions, reference sequences, and annotations were indicated according to the L. interrogans serovar Manilae strain UP-MMC-NIID LP (MicroScope Platform (https://mage.genoscope.cns.fr/microscope/home/index.php). b Isolates Id 38, 747, 806, 816, 974, 986, 1058, 1085. (B) Schematic representation of LIMLP_01895. Schematic representation and domain organization of LIMLP_01895 have been determined by ScanProsite (De Castro et al., Nucleic Ac [file ppat.1009087.s006.tif]

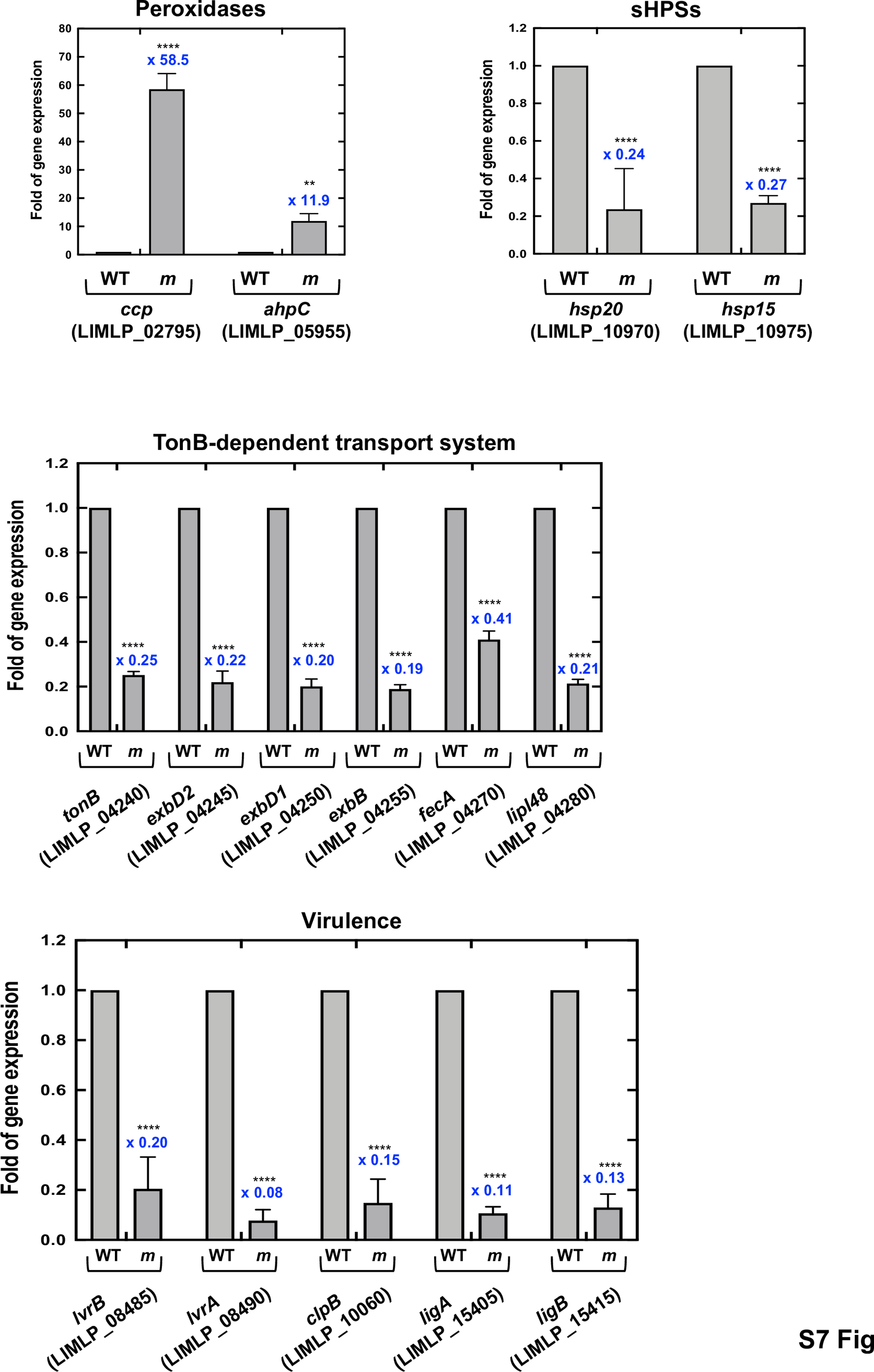

Supplement: S7 Fig — RNAs were extracted from exponentially-grown L. interrogans strains WT or double perRAperRB mutant (m). Expression of the indicated genes was measured by RT-qPCR using the LIMLP_06735 as reference gene. This gene, which encodes a protein with unknown function, was used as a reference gene since its expression was not changed upon inactivation of perRA and perRB. Gene expression in the perRAperRB mutant was normalized against that in the WT strain. Fold change values are indicated in blue. Statistical significance was determined by a Two-way Anova test in comparison with the WT samples (****, p-value<0.0001; **, p-value = 0.0048). (TIF) [file ppat.1009087.s007.tif]

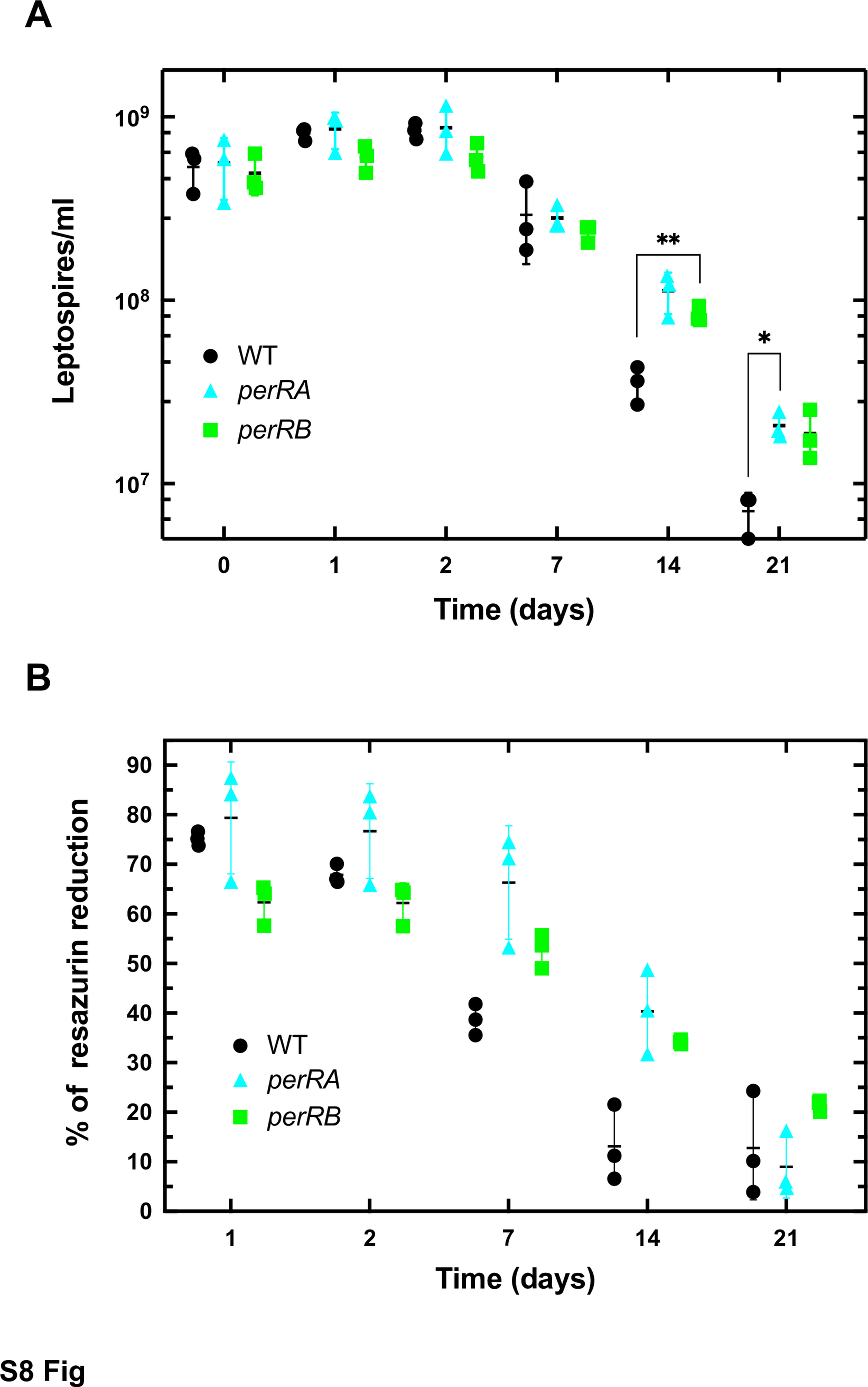

Supplement: S8 Fig — Exponentially growing WT (black circles), perRA (cyan triangles) and perRB (green squares) mutant strains were centrifugated at 2600 g for 15 min and washed three times and resuspended into filter-sterilized spring water (Volvic). All samples were adjusted to 5x10^8^ leptospires/ml. The samples were incubated at RT in darkness and, at the indicated times, leptospires were counted under a dark-field microscope using a Petroff-Hauser cell (A) and their viability was determined by quantification of resazurin reduction using the AlamarBlue reagent (ThermoScientific) (B). Data are means and standard errors of three independent biological experiments. Statistical significance was determined by a Two-way Anova test in comparison with the WT samples (**, p-value = 0.006; *, p-value = 0.0278). (TIF) [file ppat.1009087.s008.tif]
