# Supplementary material for: The oxidative stress response of pathogenic Leptospira is controlled by two peroxide stress regulators which putatively cooperate in controlling virulence
Source: PLoS Pathog. 2021 Dec 2;17(12):e1009087. doi: 10.1371/journal.ppat.1009087 (PMC8638851; doi:10.1371/journal.ppat.1009087)
Supplement: S7 Table — Significantly differentially-expressed non-coding RNAs in the perRB (M1474) mutant are listed in this Table with gene numbering according to Leptospira interrogans serovar Manilae strain UP-MMC-NIID-LP genome. (DOCX) [file ppat.1009087.s015.docx]

| **NC RNA^a^** | **Chromosome/**  **Plasmid** | **Log_2_Fc** | **Adjusted**  **p-value** | **Start-End** | **Overlapping**  **ORF** | **Upstream ORF** | **Downstream**  **ORF** |
| --- | --- | --- | --- | --- | --- | --- | --- |
| **LepncRNA35^#^ (rh753)** | NZ_CP011931.1 | -1.011 | 1.77e-09 | 602707-602840 | NA | LIMLP_02460 | LIMLP_02465 |
| **LepncRNA36** | NZ_CP011931.1 | -1.403 | 5.76e-18 | 611935-611994 | NA | LEPIMA_cI0537 | LIMLP_02490* |
| **LepncRNA87** | NZ_CP011931.1 | -1.187 | 1.15e-12 | 2078455-2078626 | NA | LIMLP_08575 | LIMLP_08580* |
| **LepncRNA89^#§^ (rh2487)** | NZ_CP011931.1 | -1.162 | 1.24e-10 | 2083793-2083898 | LIMLP_08585 | LEPIMA_cI1903 | LIMLP_08590* |
| **LepncRNA109^#§^ (rh3186)** | NZ_CP011931.1 | -1.123 | 2.06e-13 | 2658522-2658603 | NA | LIMLP_11175 | LIMLP_11180** |
| **LepncRNA139** | NZ_CP011931.1 | -1.008 | 1.17e-07 | 3459792-3459865 | NA | LIMLP_14585* | LIMLP_14590 |

**S7 Table. Selected differentially-expressed non-coding RNAs in the *perRB* mutant**

Significantly differentially-expressed predicted ncRNAs in the *perRB* mutant (M1474) with a Log_2_FC cutoff of ±1 and an adjusted p-value cutoff of 0.05.

^a^ Gene numbering is according to Satou *et al.* (1).

* ORFs significantly down-regulated by RNA-Seq analysis the *perRB* mutant (Log_2_FC cutoff of -0.5 and adj. p-value cutoff of 0.05) (this study).

** ORFs significantly up-regulated by RNA-Seq analysis the *perRB* mutant (Log_2_FC cutoff of 0.5 and adj. p-value cutoff of 0.05) (this study).

^#^ ncRNAs significantly differentially-expressed in the *perRA* mutant (M776) ((2); the corresponding name is indicated into parenthesis).

^§^ ncRNAs significantly differentially-expressed in the *perRAperRB* mutant (this study).

**References**

1. Satou K, Shimoji M, Tamotsu H, Juan A, Ashimine N, Shinzato M, et al. Complete Genome Sequences of Low-Passage Virulent and High-Passage Avirulent Variants of Pathogenic Leptospira interrogans Serovar Manilae Strain UP-MMC-NIID, Originally Isolated from a Patient with Severe Leptospirosis, Determined Using PacBio Single-Molecule Real-Time Technology. Genome Announc. 2015 Aug 13;3(4):e00882-15.

2. Zavala-Alvarado C, Sismeiro O, Legendre R, Varet H, Bussotti G, Bayram J, et al. The transcriptional response of pathogenic Leptospira to peroxide reveals new defenses against infection-related oxidative stress. PLOS Pathogens. 2020 Oct 6;16(10):e1008904.
