# Supplementary material for: The oxidative stress response of pathogenic Leptospira is controlled by two peroxide stress regulators which putatively cooperate in controlling virulence
Source: PLoS Pathog. 2021 Dec 2;17(12):e1009087. doi: 10.1371/journal.ppat.1009087 (PMC8638851; doi:10.1371/journal.ppat.1009087)
Supplement: S9 Table — The different strains used in this study are listed in this Table. (DOCX) [file ppat.1009087.s017.docx]

**Strains Description^a^ Tn insertion site^b^ Source**

____________________________________________________________________________________________________________________

*Leptospira interrogans* Wild-type strain (WT)

Serovar Manilae, strain L495

*perRA* (M766) *Himar1* Tn insertion in LIMLP_10155 (2427923-2428360) 2427985 (1,2)

*perRA::Km^R^*

Resistance to kanamycin

*perRB* (Man1474) *Himar1* Tn insertion in LIMLP_05620 (1386251-1386688) 1386423 This study *perRB::Km^R^*

Resistance to kanamycin

*perRAperRB* allelic exchange of LIMLP_10155 in the M1474 mutant 1386423 This study

Δ *perRA, perRB::Km^R^*

Resistance to kanamycin and spectinomycin

*perRB*^+^*^perRB^*  Man1474 mutant trans-complemented with the LIMLP_05620 ORF This study

contains the pNB139 plasmid

Resistance to kanamycin and spectinomycin

Π1 (Δ*thyA*) *Escherichia coli* replicative strain

β2163 (Δ*dapA*) *Escherichia coli* conjugative strain (3)

**S9 Table. Strains used in this study**

^a^ Gene name is according to *Leptospira interrogans* serovar Manilae strain UP-MMC-NIID-LP genome (4).

^b^ Tn position is according to *Leptospira interrogans* serovar Manilae strain UP-MMC-NIID-LP genome (4).

**References**

1. Murray GL, Morel V, Cerqueira GM, Croda J, Srikram A, Henry R, et al. Genome-Wide Transposon Mutagenesis in Pathogenic Leptospira Species. Infect Immun. 2009 Feb 1;77(2):810–6.

2. Lo M, Murray GL, Khoo CA, Haake DA, Zuerner RL, Adler B. Transcriptional response of Leptospira interrogans to iron limitation and characterization of a PerR homolog. Infect Immun. 2010 Nov;78(11):4850–9.

3. Demarre G, Guérout A-M, Matsumoto-Mashimo C, Rowe-Magnus DA, Marlière P, Mazel D. A new family of mobilizable suicide plasmids based on broad host range R388 plasmid (IncW) and RP4 plasmid (IncPα) conjugative machineries and their cognate Escherichia coli host strains. Research in Microbiology. 2005 Mar 1;156(2):245–55.

4. Satou K, Shimoji M, Tamotsu H, Juan A, Ashimine N, Shinzato M, et al. Complete Genome Sequences of Low-Passage Virulent and High-Passage Avirulent Variants of Pathogenic Leptospira interrogans Serovar Manilae Strain UP-MMC-NIID, Originally Isolated from a Patient with Severe Leptospirosis, Determined Using PacBio Single-Molecule Real-Time Technology. Genome Announc. 2015 Aug 13;3(4):e00882-15.
