# Supplementary material for: The oxidative stress response of pathogenic Leptospira is controlled by two peroxide stress regulators which putatively cooperate in controlling virulence
Source: PLoS Pathog. 2021 Dec 2;17(12):e1009087. doi: 10.1371/journal.ppat.1009087 (PMC8638851; doi:10.1371/journal.ppat.1009087)
Supplement: S10 Table — The different plasmids used in this study are listed in this Table. (DOCX) [file ppat.1009087.s018.docx]

**Plasmids Description** ^a^ **Source**

pMaORI replicative and conjugative vector for *Leptospira* (1)

pNB139 pMaORI containing the LIMLP_05620 (*perRB*) ORF This study

Promoter: 200 bp upstream region

Resistance to spectinomycin

pKΔperRA suicide plasmid containing a spectinomycin resistance cassette This study

flanked by the LIMLP_10155 (*perRA*) ORF

**S10 Table. Plasmids used in this study**

^a^ Gene name is according to *Leptospira interrogans* serovar Manilae strain UP-MMC-NIID-LP genome (2).

**References**

1. Pappas CJ, Benaroudj N, Picardeau M. A Replicative Plasmid Vector Allows Efficient Complementation of Pathogenic Leptospira Strains. Parales RE, editor. Appl Environ Microbiol. 2015 May 1;81(9):3176.

2. Satou K, Shimoji M, Tamotsu H, Juan A, Ashimine N, Shinzato M, et al. Complete Genome Sequences of Low-Passage Virulent and High-Passage Avirulent Variants of Pathogenic Leptospira interrogans Serovar Manilae Strain UP-MMC-NIID, Originally Isolated from a Patient with Severe Leptospirosis, Determined Using PacBio Single-Molecule Real-Time Technology. Genome Announc. 2015 Aug 13;3(4):e00882-15.
